# Spatiotemporal Properties of Common Semantic Categories for Words and Pictures

**DOI:** 10.1162/jocn_a_02182

**Published:** 2024-07-01

**Authors:** Yulia Bezsudnova, Andrew J. Quinn, Syanah C. Wynn, Ole Jensen

**Affiliations:** 1 University of Birmingham; 2 Gutenberg University Medical Center Mainz

## Abstract

◼The timing of semantic processing during object recognition in the brain is a topic of ongoing discussion. One way of addressing this question is by applying multivariate pattern analysis to human electrophysiological responses to object images of different semantic categories. However, although multivariate pattern analysis can reveal whether neuronal activity patterns are distinct for different stimulus categories, concerns remain on whether low-level visual features also contribute to the classification results. To circumvent this issue, we applied a cross-decoding approach to magnetoencephalography data from stimuli from two different modalities: images and their corresponding written words. We employed items from three categories and presented them in a randomized order. We show that if the classifier is trained on words, pictures are classified between 150 and 430 msec after stimulus onset, and when training on pictures, words are classified between 225 and 430 msec. The topographical map, identified using a searchlight approach for cross-modal activation in both directions, showed left lateralization, confirming the involvement of linguistic representations. These results point to semantic activation of pictorial stimuli occurring at ~150 msec, whereas for words, the semantic activation occurs at ~230 msec. ◼

## Introduction

Humans are capable of recognizing and inferring the semantic category of a presented object regardless of the modality in which it is presented, whether through visual, auditory, or textual means.

A powerful way of studying how the human brain encodes the semantics of objects is to apply multivariate pattern analysis (MVPA) to human electrophysiological responses to stimuli of different conceptual categories (Groen, Dekker, Knapen, & Silson, 2022; Liuzzi, Aglinskas, & Fairhall, 2020; Cichy, Pantazis, & Oliva, 2014; Carlson, Tovar, Alink, & Kriegeskorte, 2013). The timing of the transition from visual representations of individual objects to more abstract semantic-type concepts is determined by identifying the time points at which the category of the object can be accurately predicted from the multivariate representation in the magnetoencephalography (MEG) or EEG data (Wardle & Baker, 2020; Wang, Kuperberg, & Jensen, 2018; Contini, Wardle, & Carlson, 2017; Kumar, Federmeier, Fei-Fei, & Beck, 2017; Proklova, Kaiser, & Peelen, 2016). However, drawing meaningful conclusions from the above-chance classification using stimuli from a single modality may be limited, as there are often multiple dimensions in which the two conditions differ (Frisby, Halai, Cox, Ralph, & Rogers, 2023; Peelen & Downing, 2023). For example, one can argue that perceptual information beyond semantic content influences classification outcomes. Furthermore, the temporal dynamic of object recognition may also be influenced by the specific experimental task employed in the study, such as a one-back task, category judgment, or detection task. As such, the precise timing of semantic category activation remains an open question (Dirani & Pylkkänen, 2020, 2023; Giari, Leonardelli, Tao, Machado, & Fairhall, 2020; Kaiser, Azzalini, & Peelen, 2016; Miozzo, Pulvermüller, & Hauk, 2015; MacGregor, Pulvermüller, Van Casteren, & Shtyrov, 2012; Simanova, Van Gerven, Oostenveld, & Hagoort, 2010).

Studying brain responses to stimuli from different modalities (e.g., words vs. images) mitigates the criticism related to the influence of perceptual features on classification, as these stimuli do not share common low-level features (Hauk, Davis, Ford, Pulvermüller, & Marslen-Wilson, 2006). In addition, demonstrating the existence of cross-modal generalization (training a classifier on one modality and classifying another) at specific time points will provide insights into the ongoing debate about how and when semantic information is encoded (Dirani & Pylkkänen, 2023; Federmeier, 2022; Hauk, Coutout, Holden, & Chen, 2012; Indefrey, 2011).

In studies involving image recognition, findings indicate that low-level perceptual features are activated within the first 100 msec, followed by the emergence of representations related to semantic categories (e.g., animal) by 150 msec (Miozzo et al., 2015; Cichy et al., 2014; Carlson et al., 2013; Clarke, Taylor, Devereux, Randall, & Tyler, 2013). However, as mentioned earlier, it is debated to which extent perceptual features influence the time course (Proklova, Kaiser, & Peelen, 2019). The timing of semantic activation in the textual modality has been investigated, and it is evident that low-level features of written words do not contain information about the semantic category (Dirani & Pylkkänen, 2023; Federmeier, 2022; Hauk et al., 2012; Indefrey, 2011). Some studies assert that semantic processing occurs at approximately 300– 500 msec after stimulus onset, as indicated by the N400 response (Grainger & Holcomb, 2009; Pylkkänen & Marantz, 2003). Others argue that information becomes available in a graded manner following stimulus onset, and the N400 serves as an indicator of postcomprehension processes associated with the semantic integration of stimuli into the context (Federmeier, 2022; DeLong & Kutas, 2020; Giari et al., 2020; Pulvermüller, Shtyrov, & Hauk, 2009). These arguments are based on studies pointing to components of semantic processing occurring around as early as 150–200 msec (Giari et al., 2020; Amsel, Urbach, & Kutas, 2013; Hauk et al., 2012; Amsel, 2011; del Prado Martín, Hauk, & Pulvermüller, 2006; Thorpe, Fize, & Marlot, 1996). Finding evidence for semantic representation shared across modalities would contribute to an improved understanding of the timeline of semantic activation but would also be aligned with the hub-and-spoke theory of semantic representations, where modality-specific representations interact via an amodal hub (Ralph, Jefferies, Patterson, & Rogers, 2017; Humphreys, Hoffman, Visser, Binney, & Lambon Ralph, 2015; Pobric, Jefferies, & Ralph, 2007). There is limited research done investigating the time of the common cross-modal generalization using M/EEG, and the reported temporal dynamic of cross-modal representation varies (Dirani & Pylkkänen, 2023; Iamshchinina, Karapetian, Kaiser, & Cichy, 2022; Giari et al., 2020; Leonardelli, Fait, & Fairhall, 2019; Simanova et al., 2010).

In one MEG study (Leonardelli et al., 2019), the categorization between famous places (*“*Big Ben*”*) and famous people (*“*Brad Pitt*”*) is studied in the picture and written word modalities. After each stimulus, participants were asked to perform either a shallow categorization task (Place or Person?) or a deeper semantic task (*“*Italian or foreign?*”*). Within the modality (modality-specific), category information robustly appears around 100 msec for pictures and 230 msec for words. The cross-modal (across modality) classification was studied only from words to pictures and revealed three significant clusters separated in time. Specifically, the authors suggest that cross-modal generalization unfolds through a three-stage process and the first shared representations are accessed at 200 msec using words and at 110 msec using pictures. However, brain activity from concepts such as famous places and people used in their paradigm might not generalize to more common objects. Furthermore, any findings of cross-modal representations across modalities could be driven by a shared category-judgment process because of a category-naming task rather than an automatic activation of semantic representations (Hebart, Bankson, Harel, Baker, & Cichy, 2018).

Another recent MEG study (Dirani & Pylkkänen, 2023) examining generalization between written words and pictures, using picture naming and word reading tasks, showed different results. Significant decoding (animal vs. tool) activates surprisingly early around 75 msec for pictures, and 95 msec for words. Cross-generalization occurs simultaneously for both modalities around 150 msec. The different time courses of the shared semantic representation between two studies (Dirani & Pylkkänen, 2023; Leonardelli et al., 2019) could be attributed to variations in participant tasks and experimental paradigms. Importantly, in the latter study (Dirani & Pylkkänen, 2023), a block presentation of categories was utilized to demonstrate cross-modal categorization. This might result in anticipatory effects making the semantic categorization occur earlier. On the basis of these considerations, the precise timing of the activation of the shared representation should be reevaluated in experiments where the category order is randomized while explicit category naming is not required.

In this work, we investigated the time course of semantic activation within and across pictural and textual stimulation presented in a randomized order. We analyzed MEG data using MVPA. To further eliminate any anticipatory biases, such as the possibility of stronger activation of general motor commands for the tools category, we chose to include multiple categories. Finally, we applied a search-light approach to identify the brain areas involved in the semantic representations.

## Methods

### Participants

Thirty-eight healthy adult participants took part in the study. Five participants had to be excluded because of extensive noise in the data, and two participants were excluded because of low accuracy during the task (accuracy less than 85%). Therefore, the final sample consisted of 31 participants (mean age = 21.77 years, *SD* = 3.31 years; 21 female participants). The number of participants was selected to match the average number of participants used in previous studies that employed MVPA (Dirani & Pylkkänen, 2023; Singer, Cichy, & Hebart, 2023; Leonardelli et al., 2019). The study was conducted at the Center for Human Brain Health in Birmingham, United Kingdom. All participants were native English speakers with normal or corrected-to-normal vision. The University of Birmingham Ethics Committee approved the study. The participants were provided written informed consent and received either £15 per hour or course credits as compensation for their participation.

### Experimental Paradigm

The experimental design used here was an adapted version of the experimental paradigm used in a previous study (Iamshchinina et al., 2022). However, some stimuli were replaced and the task for the participant was modified. The stimulus set was composed of 48 objects. Each stimulus was presented as a picture and a written word. The objects were organized according to three dimensions of categories, each separated into two categorical divisions: *“*size*”* (big or small), *“*movement*”* (mobile or still), and *“*nature*”* (natural or man-made). Each object in the study belonged to each of the dimensions (e.g., a book is small, still, and man-made). The stimulus set was balanced such that each categorical division included one half of the stimulus set (24 objects). Hence, each set of categories (e.g., big, still, natural) included six objects. The selection of categorical divisions in this study was based on previous findings demonstrating reliable neural representations of semantic dimensions across categories (Iamshchinina et al., 2022; Konkle & Caramazza, 2013). Each object was presented in two modalities: written words and pictures. The size of the images was 400 × 400 pixels presented on a gray screen at a visual angle of 6°. In the stimuli set for textual modality (font: Arial, bold, 75), we did not control for the lexical properties of the words, which resulted in a mixture of high-frequency and low-frequency words. In addition, the length of the words was not controlled. Importantly, we did not find any significant differences (two-tailed *t* test, *p* > .3) in word frequency or word length obtained via The English Lexicon Project (Balota et al., 2007) across any pair of categorical divisions, namely, *“*size,*” “*movement,*”* and *“*nature.*”*

**Figure 1:**
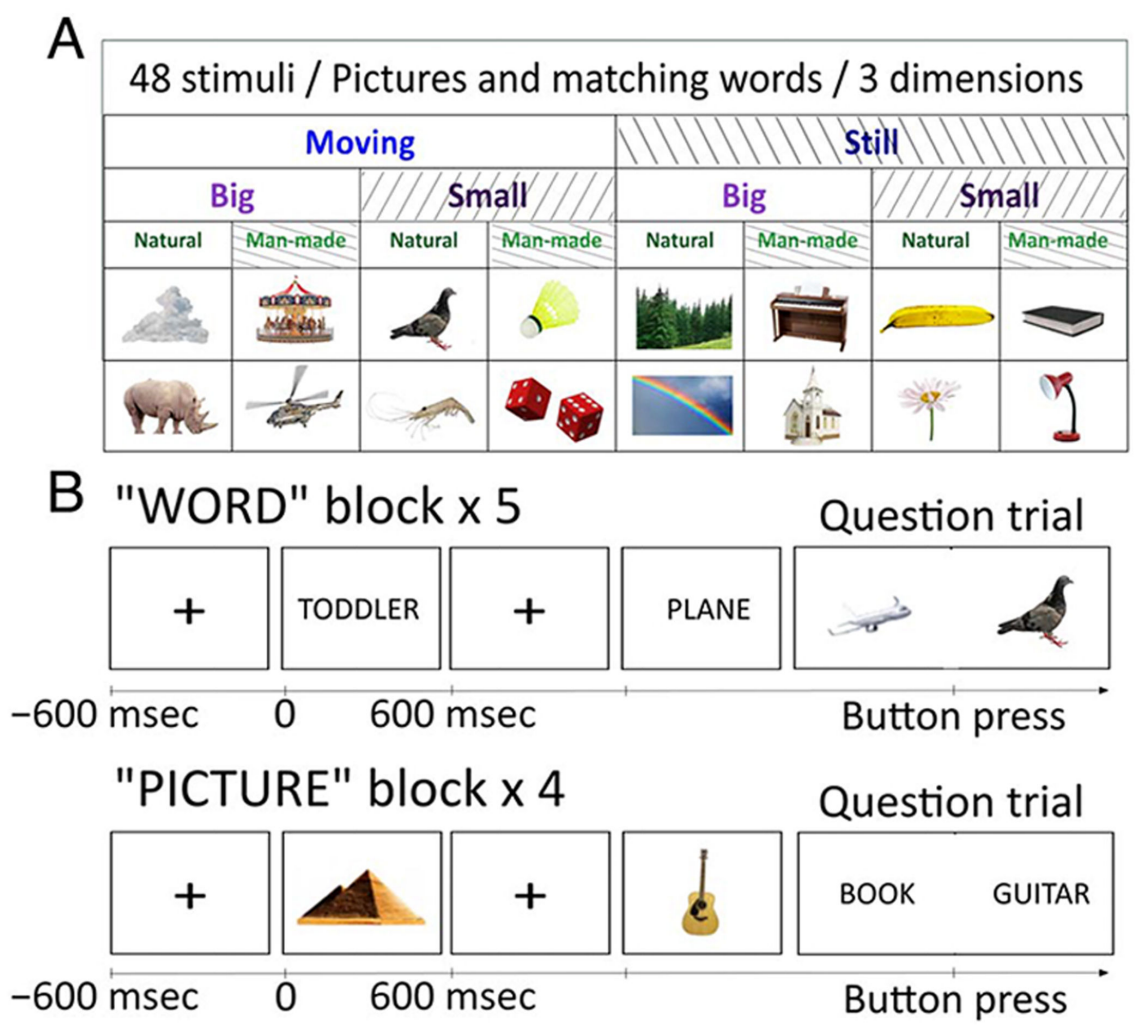
Experimental paradigm. (A) The stimulus set is composed of 48 objects that can be divided according to three dimensions (*“*size*”*: big/small; *“*movement*”*: still/mobile; *“*nature*”*: natural/man-made). (B) In both image and word blocks, participants were presented with stimuli in random order. In the picture block, participants viewed images of the objects, whereas in the word block, they read the corresponding words. The task (question trial) was presented randomly every fifth trial on average. Participants were required to identify whether the picture or word corresponded to a previously seen stimulus and press the appropriate button accordingly. When pictures were presented, the probe question was presented as a word and vice versa.

Each object (word or picture) was presented for 600 msec. Before the object presentation, the fixation cross was shown for 500–700 msec. The experiment was divided into nine blocks where either words (five blocks) or images (four blocks) were successively presented. The experimental design followed a consistent order, with word blocks always presented first, followed by image blocks, and so on (Figure 1B). After each block, a participant had a break. We included an extra word block based on previous reports that indicated a lower signal-to-noise ratio for classification in the textual modality (Dirani & Pylkkänen, 2023; Simanova et al., 2010). Each block consisted of 240 trials, with 48 of them being question trials, and lasted for around 6 min. Each stimulus was present 4 times in a block. During a probe question, two stimuli were displayed on the screen with one of them corresponding to the previously presented stimuli. Participants were asked to press either the left or right button to identify it. For image blocks, probe questions were presented in word modality and vice versa (Figure 1B). As such, a participant had to identify the correct picture during the written words and vice versa. This design aimed to keep participants engaged and stimulate deep cross-modal perception without explicitly addressing the category distinction between stimuli. Note that in each block the probe question was applied for every object once and alternative choices were unique as well.

The stimuli presentation was implemented in MATLAB (The MathWorks) using the Psychophysics Toolbox (Brainard, 1997).

### MEG Acquisition

MEG data were recorded using a 306-sensor TRIUX MEGIN Elekta Neuromag system, consisting of 204 orthogonal gradiometers and 102 magnetometers, with online band-pass filtering from 0.1 to 330 Hz and a sampling rate of 1000 Hz. Before collecting data, the positions of three anatomical fiducial points (nasion, left preauricular, and right preauricular points) were recorded using a Polhemus Fastrack electromagnetic digitizer system. Furthermore, we recorded the positions of four head-position indicator coils: two placed on the left and right mastoid bone, and two on the forehead, with a minimum separation of 3 cm between each coil. After completing these initial steps, participants were seated in an upright position with a 60° angled backrest within the MEG gantry. Electrodes were affixed about 2.5 cm from the outer canthus of each eye to capture horizontal EOG signals. To record the VEOG, a pair of electrodes was placed above and below the right eye in line with the pupil. The electrocardiography signals were captured using a set of electrodes positioned on both the left and right collarbones.

### Data Preprocessing

The data are analyzed using the open-source toolbox MNE Python v1.4.2 (Gramfort et al., 2013) following the standards defined in the FLUX Pipeline (Ferrante et al., 2022).

First, blinks and muscle artifacts are annotated using EOG channels and magnetometers recordings, respectively. A semi-automatic detection algorithm was utilized to mark sensors with excessive artifacts (on average 4.5 channels per participant). Then, the data were low-pass filtered at 100 Hz to reduce head-position indicator coils artifacts. We did not use signal space separation (SSS) or Maxwell filtering (Taulu & Simola, 2006) because our previous research demonstrated that they negatively impact classification results (in preparation; Bezsudnova & Jensen, 2023). We attribute this decline in performance to the increase of the white noise in the data following SSS filtering. For a more detailed description of how the SSS filter amplifies the white noise in the data, please refer to Taulu, Simola, and Kajola (2005). Signal Space Projection (eight system-provided projectors) and then independent component analysis (ICA) algorithms were applied (Hyvärinen & Oja, 2000; Uusitalo & Ilmoniemi, 1997) to remove external interference and components associated with cardiac artifacts and eyeblinks. The identification process involved analyzing the time courses and topographies of the ICA components. On average, three components corresponding to two cardiac-related and one blink-related artifact were identified and removed for each subject. After ICA, the data were segmented into trials. Trials corresponding to the same stimulus were averaged together to construct *“*super-trials.*”* This step further enhances the signal-to-noise ratio of the data (Ashton et al., 2022; Guggenmos, Sterzer, & Cichy, 2018). We down-sampled the data to 500 Hz. The epochs were time-locked to the onset of the stimuli and were cropped to a time window of 100 msec before the stimuli and 700 msec after the stimuli onset. Each time point was represented as a 306-dimensional vector (channel data). We expanded this vector by including past 25 msec (12 time points) and future 25 msec (12 time points) of data, resulting in a 306 × 25 dimensional feature vector representing each time point. This procedure (termed *“*delayed embedding*”*) resulted in a more information-rich representation of the neural activity associated with a given stimulus by incorporating more time points (Cheng, 2021; Grootswagers, Wardle, & Carlson, 2017; Tyler, Cheung, Devereux, & Clarke, 2013; Chan, Halgren, Marinkovic, & Cash, 2011). Therefore, the feature vector is constructed from super-trials with embedded 50 msec of data. As the last step before applying the classifier to the data, the feature vectors were standardized by removing the mean and scaling to unit variance per sensor. Following this procedure, the data from gradiometers and magnetometers can be combined.

### Classification Analysis

MVPA was applied to the preprocessed MEG data to classify the categories of the presented objects (Cichy et al., 2014; Carlson et al., 2013; Haynes & Rees, 2006). The feature vectors associated with different categories (e.g., mobile vs. still) and one modality (e.g., words) are labeled accordingly and used as input for the classifier. We employed a support vector machine (Cortes & Vapnik, 1995) from the Python module Scikit-learn to classify the data over time. The classification procedure relied on a fivefold cross-validation approach, and the performance was quantified using the area under the curve metric, which measures the classifier discriminative ability. This procedure was done separately for each subject.

First, we investigated the classification accuracy for each category dimension (*“*movement,*” “*size,*”* and *“*nature*”*) averaged across participants for each modality separately. The dimensions where both modalities showed a classification accuracy significantly above the chance level were selected for further analysis. We averaged the classification curves of the chosen categorical dimensions to obtain results that were less contaminated by low-level features (Iamshchinina et al., 2022). The same categorical dimensions were selected for cross-modal analysis. For cross-modal analysis, we also used time-generalized MVPA (King & Dehaene, 2014). We used the classification approach mentioned above with the exception that the classifier was training in one modality at one time point and testing it on another at a different time point. This procedure was done twice, once with the classifier trained on the words data and tested on the pictures data, and once where it was trained on the pictures data and tested on the words.

To explore the spatial distribution of representations across the MEG sensors over time, we employed the searchlight approach on sensor-level data (Leonardelli et al., 2019; Kriegeskorte, Mur, & Bandettini, 2008). In this approach, classification was performed on *“*patches*”* that are defined by all sensors within a 4-cm radius from a specific sensor (e.g., 4 cm from MEG 1423). This typically resulted in 15 sensors (consisting of gradiometers and magnetometers). Therefore, the feature vector had dimensions *N* × 25, where *N* is the number of sensors in the *“*patch*”* and 25 are time points from the delayed-embedding procedure. Patches were created for all possible sensor locations (rim sensors had fewer sensors in the patch). For each channel location, the classification accuracy from the relevant *“*patch*”* was averaged over a chosen time interval and this value was plotted on the topographical sensor map.

### Statistical Analysis

To find significant time points for the modality-specific classification curves and simultaneous cross-generalization, we used a nonparametric, one-sampled permutation *t* test (one-tailed) against 50% chance level, controlled for multiple comparisons (Sassenhagen & Draschkow, 2019; Maris & Oostenveld, 2007) implemented in the GLMTools Python package (https://pypi.org/project/glmtools/). We set a cluster-forming threshold of 1.7, corresponding to an alpha threshold of .05. Clusters of *t* values that exceeded the cluster-forming threshold were formed based on direct adjacency in time (the minimum number of vertices in terms of time points in a cluster was set to 2) and summarized using the sum of *t* values within the cluster. The largest cluster from each of the *N* sign-flip permutations were computed to form a null distribution. A cluster in the observed *t* values was considered significant if its cluster stat (sum of *t* value with cluster) lay at or above the 95 th percentile of the null distribution. This corresponds to an alpha threshold of *p* = .05.

The same analysis was done for cross-modal time generalization results. Note that in this case, the clusters are based on direct adjacency in both axes.

## Results

### Within-modality Decoding

When considering pictures, we find robust decoding for every categorical dimension (*“*size,*” “*movement,*”* and *“*nature*”*) separately. However, for words, we found robust classification only for dimensions *“*size*”* (big/small) and *“*movement*”* (still/mobile), but not *“*nature*”* (natural/ man-made). In a previous study (Cichy et al., 2014), this category dimension also exhibited the lowest decoding accuracy when examining pictures. Other modalities such as written or spoken words have generally shown lower classification accuracy compared with pictures (Dirani & Pylkkänen, 2023; Iamshchinina et al., 2022), suggesting that the *“*nature*”*-dimension in textual modality would be very difficult to classify. We also speculate that the *“*nature*”* category we applied was too broad. We included any nature objects such as *“*mountain,*” “*rainbow,*”* and *“*forest*”* rather than, for example, more specific and standard objects from the animal world. All the results presented were therefore derived by averaging the classification results obtained from just two categorical dimensions (*“*size*”* and *“*movement*”*).

**Figure 2:**
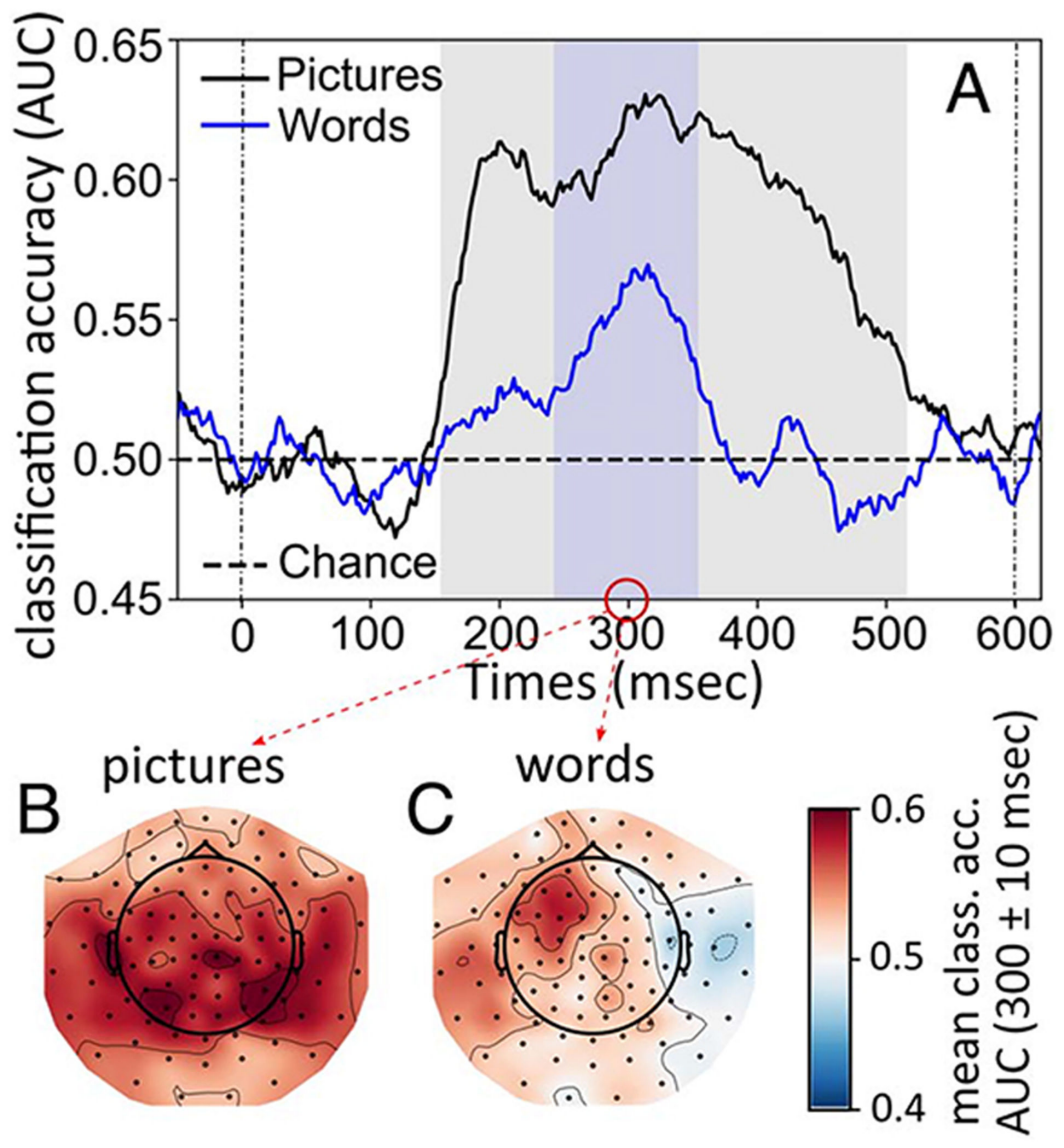
(A) The time course of category decoding averaged over two categorical dimensions: *“*size*”* (big/small), and *“*movement*”* (still/ mobile). The blue line marks the classification curve for word modality; the black line marks the classification curve for picture modality. Significant clusters (*p* < .05; controlled for multiple comparisons over time) are shown as highlighted areas accordingly. (B) The topographical map of category decoding averaged over 300 ± 10 msec created using searchlight MVPA decoding categories of pictures. (C) Decoding categories of words. The colorcode indicates the classification accuracy (area under the curve) averaged over the time interval 300 ± 10 msec. The classification accuracy is averaged over two category dimensions: *“*size*”* (big/small) and *“*movement*”* (still/mobile).

The classification accuracies for the picture modality and textual modality are shown in Figure 2A. As expected, a classifier that uses brain activity elicited by words showed lower performance compared with picture stimuli (Ghazaryan et al., 2023). For words, decoding is most pronounced around 240–350 msec after stimuli are presented. For pictures, decoding is most pronounced around 155–510 msec. These results indicate that semantic activation for words occurs approximately 100 msec later than for pictures. Note that the time estimation might be blurred because of the feature vector including ±25 msec of information; however, the relative relationships between modalities are preserved.

For the time points (300 ± 10 msec) when the decoding accuracy is strongest, we show topographical maps of the classification accuracy using a searchlight approach. The sensors that contributed strongest to the overall accuracy of the picture category are located over bilateral temporal and parietal areas (Figure 2B). For the decoding word category, the informative sensors are located over the left temporal and left frontal parts of the brain (Figure 2C). This localization is in line with prior results using MEG in object decoding (Cichy et al., 2014) and word reading studies (Hultén et al., 2021; Kumar et al., 2017; Santi, Friederici, Makuuchi, & Grodzinsky, 2015).

### Cross-modal Decoding

**Figure 3:**
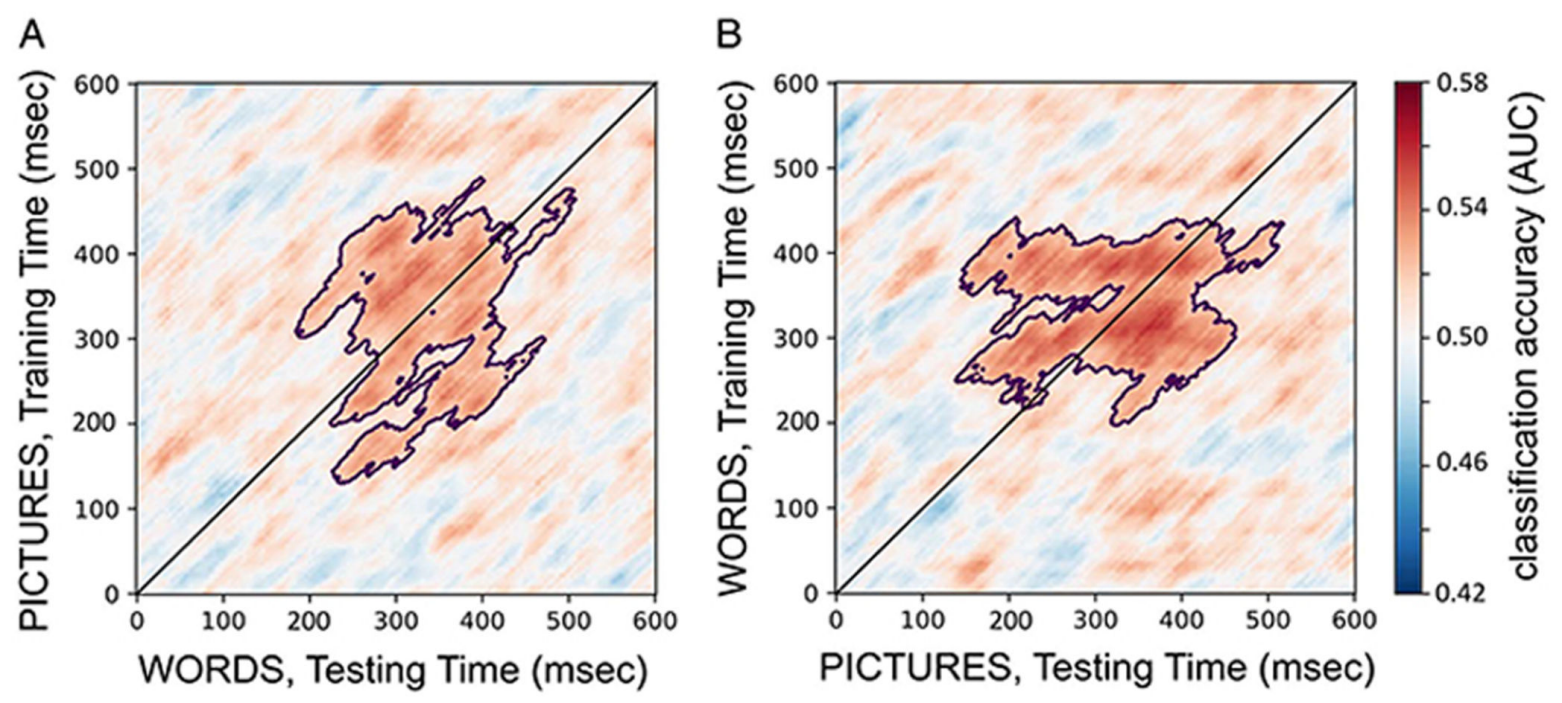
Time generalization results for category information, where classifier was (A) trained on pictures and tested on words, and (B) trained on words and tested on pictures. The classification accuracy is averaged over two categorical dimensions: *“*size*”* (big/small) and *“*movement*”* (still/ mobile). Significant clusters are highlighted (one-sided permutation test, *p* < .05, corrected for multiple comparisons).

Time-generalized, cross-decoding results is shown in Figure 3. When training on pictures and testing on words, shared representation occurs between 225 and 430 msec (Figure 3A); when training on words and testing on pictures, shared representation occurs between 150 and 430 msec (Figure 3B). Interestingly, the most pronounced semantic decoding across modalities for both words and pictures becomes significant around the same time as the categories are classified within each modality (Figure 2A).

**Figure 4:**
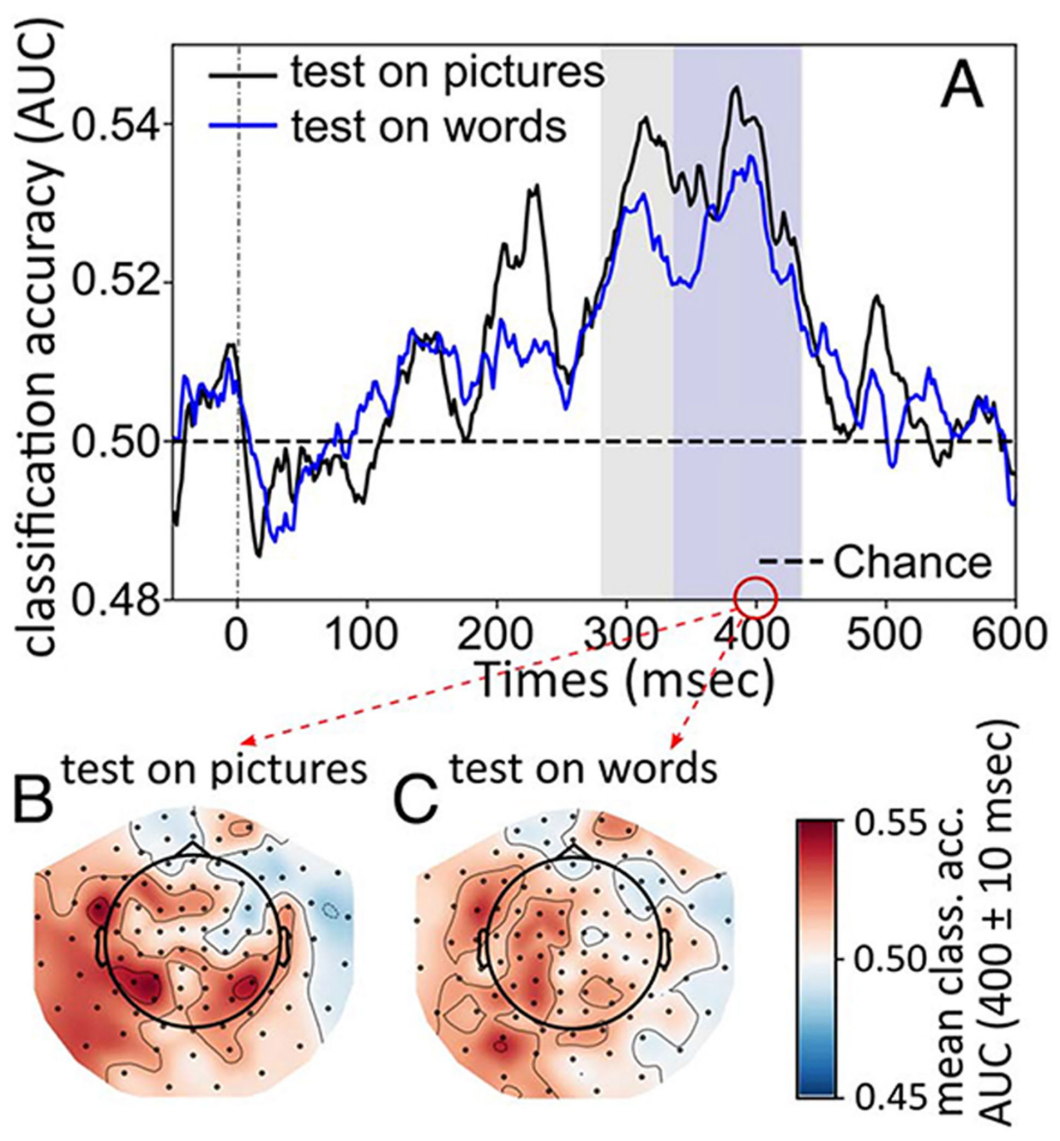
(A) The time course of simultaneous (trained and tested on the same time points) cross-modal category decoding averaged over two categorical dimensions: *“*size*”* (big/small) and *“*movement*”* (still/ mobile). The blue line marks the classification curve when classifiers were trained on pictures and tested on words; the black line marks the classification curve when trained on words and tested on pictures. Significant clusters (*p* < .05; controlled for multiple comparisons over time) are shown as highlighted areas accordingly. (B) Topographical map of cross-modal categorical decoding averaged over 400 ± 10 msec created using searchlight MVPA: when training on words, testing on pictures modality; (C) when training on pictures, testing on words modality. The colorcode indicates the classification accuracy averaged over the time interval 400 ± 10 msec. The classification accuracy is averaged over two categorical dimensions: *“*size*”* (big/small) and *“*movement*”* (still/mobile).

In addition, we showed the simultaneous (trained and tested on the same time points) cross-modal classification between textual and pictural modality in Figure 4A. When training on words and testing on pictures, the significant cluster emerged at 280–430 msec. When reversely training on pictures and testing on words, the cluster emerged at 330–430 msec.

Next, we examined the topographical maps of the decoding accuracy at 400 ± 10 msec to check where the simultaneous cross-modal classification is the most pronounced (Figure 4B and C). We selected the time interval of 400 ± 10 msec because it corresponds to the peak decoding accuracy in Figure 4A. These maps for simultaneous cross-modal activation in both directions show more pronounced left lateralization (Figure 4B and C) compared with topographies from within-modality decoding (Figure 2B and C). Activation in the inferior parietal cortex is in line with Liu, Wang, Zhou, Ding, and Luo (2017).

In summary, when considering the within- and cross-modal results, this point to semantic activation of pictures and words occurs at ~150 msec and ~230 msec, respectively.

## Discussion

In this study, we investigated the temporal dynamics of semantic processing when objects were presented as pictures and text. We assessed the qualitative similarities between categories elicited for each modality using MVPA on MEG data. The classification shows the most pronounced decoding activation for the pictures starting at 155 msec over the bilateral posterior part of the brain and for words after 240 msec over the left temporal and frontal parts of the brain (Figure 2). We show successful cross-modal classification using MEG data (Figure 3). If the classifier is trained on words, pictures are classified between 150 and 430 msec, and when training on pictures, words are classified between 225 and 430 msec. The topographical map for simultaneous cross-modal activation in both directions (from words to pictures and from pictures to words) reveals strong left lateralization (Figure 4B and C).

Semantic activation for pictures starts around 150 msec, consistent with findings reported in a previous study (Iamshchinina et al., 2022) that employed a similar set of stimuli. The temporal dynamics of representations elicited by words demonstrate a later activation around 240 msec, which is in line with Giari and colleagues (2020) and Leonardelli and colleagues (2019) and studies using the N400 paradigm where the semantic response starts to build up at 250 msec (Federmeier, 2022; Giari et al., 2020; Amsel et al., 2013; Hauk et al., 2012). The different timing of picture and word categorization can be explained by the fact that low-level visual features in written words are less indicative of semantic categories compared with pictures (Hauk et al., 2006; Job & Tenconi, 2002). Therefore, words may activate categorical information through different more time-consuming processes from those elicited by pictures (Dirani & Pylkkänen, 2023; Indefrey, 2011; Job & Tenconi, 2002). Note that decoding onsets of 75 msec for pictures and 95 msec for words acquired in the study (Dirani & Pylkkänen, 2023) is almost 70 msec earlier than what we have demonstrated here. This disparity could likely be attributed to the categorical presentation in blocks as in the study by Dirani and colleagues (Dirani & Pylkkänen, 2023), which facilitates faster category extraction.

The time course of cross-modal decoding (Figure 3) with off-diagonal time points structured as a rectangle suggests the shared semantic representation exhibits some sustained features common to both modalities. For word modality, for example, when training on pictures and testing on words (Figure 3A), sustained shared semantic representation emerges in the 225- to 430-msec interval, whereas for pictures, for example, when training on words and testing on pictures (Figure 3B), the shared representation emerges in 150- to 430-msec interval. The delayed semantic activation prompted by words compared with pictures is also evident in the within-modality classification results shown in Figure 2. We attribute this to words having abstract low-level orthographic features that are not informative about category assignment (Hauk et al., 2006). Therefore, words require orthographical processing before their semantics can be accessed (Dirani & Pylkkänen, 2023; Giari et al., 2020; Hauk et al., 2012; Pulvermüller et al., 2009) and the categorical information is activated slower than for pictures.

In summary, both within-modality classification results and cross-modal classification results show the difference in the time course of categorical classification between pictures and words, indicating the semantics extracted through different processes for pictures compared with words (Dirani & Pylkkänen, 2023; Federmeier, 2022; Indefrey, 2011).

As mentioned in the introduction of this article, the perception and representation of the stimuli might be influenced by the experimental paradigm. Tasks relying on 1-back comparisons may not elicit strong semantic processing of the stimuli, possibly explaining why a significant cross-modal classification was not found in a previous study (Iamshchinina et al., 2022). In a study (Giari et al., 2020) where no cross-modal decoding was found, the task forced subjects to focus on the similarity of the objects by rating them according to a broader category. This might not have elicited sufficiently deep conceptual processing of the images. In our experiment, participants had to identify the word that corresponds to the previously seen picture and vice versa; therefore, the association between the two modalities was encouraged. We acknowledge that this procedure serves to promote cross-model activation. The relationship between association or mental imagery and semantic activation remains an open question for future research (Xie, Kaiser, & Cichy, 2020; Shatek, Grootswagers, Robinson, & Carlson, 2019). We attribute comparable timing of modality-specific and cross-modal semantic decoding to linguistic activation when viewing both pictures and words because of the task design. As a next step, it would be interesting to explore paradigms using unique stimuli instead of repeated images of the objects to control for the pairing accumulating between words and corresponding pictures.

Linguistic activation when looking at both pictures and words also explains why the topographical maps from cross-modal decoding for both directions (Figure 4B and C) are located over the left regions of the brain, resembling the topographical map of word categorization. Words elicit abstract shared category representations because of their nonrepresentative, low-level feature, whereas categorization within pictorial modality is contaminated by perceptual features. Furthermore, the location of most informative sensors may include the left anterior temporal lobe (ATL) as shown with fMRI to be involved in semantic processing across various tasks and including cross-modal generalization between written words and corresponding images (Branzi, Humphreys, Hoffman, & Ralph, 2020; Humphreys et al., 2015). These studies support the hub- and-spoke theory (Ralph et al., 2017) in which the ATL supports shared representations. Although our MEG study is inconsistent with the hub-and-spoke theory for semantic representations, our topographical plots do point to an extended network supporting the semantic encoding going beyond the ATL. In the future, it would be interesting to investigate the role of each frequency band in the development of semantic representation (Xie et al., 2020; Bastos et al., 2015).

## Conclusion

Our results demonstrate the time course of modality-independent semantic representations isolated from perceptual confounds. Specifically, we examined the time course of activation of semantic representations common for words and pictures. We found that the semantic activation of words occurs at ~230 msec, and for pictures, it occurs at ~150 msec. In the future, we will conduct the same experiment with an optically pumped magnetometer–MEG system to check our hypothesis that the new system can offer significant advantages in experiments designed for MVPA.

## Data Availability

Raw data are available upon request, and code can be accessed on https://github.com/Y-Bezs/cross-modal-project.
